# Is Insulin Resistance an Independent Predictor of Atherosclerosis?

**DOI:** 10.3390/jcm14030969

**Published:** 2025-02-03

**Authors:** Małgorzata Landowska, Bernadetta Kałuża, Cezary Watała, Emilia Babula, Aleksandra Żuk-Łapan, Kinga Woźniak, Aleksandra Kargul, Jonasz Jurek, Tomasz Korcz, Małgorzata Cicha-Brzezińska, Edward Franek

**Affiliations:** 1 Department of Internal Medicine, Endocrinology and Diabetology, National Medical Institute of the Ministry of the Interior and Administration in Warsaw, 02-507 Warsaw, Poland; malgorzata.landowska97@wp.pl (M.L.); edward.franek@cskmswia.gov.pl (E.F.); 2 Students Scientific Group of the Medical University of Warsaw at the Department of Internal Medicine, Endocrinology and Diabetology, National Medical Institute of the Ministry of the Interior and Administration in Warsaw, 02-507 Warsaw, Poland; emilia.babula@wum.edu.pl (E.B.); aleksandrazuk11@gmail.com (A.Ż.-Ł.); kinga966@outlook.com (K.W.); aleksandra.k96@gmail.com (A.K.); jurekjonasz@gmail.com (J.J.); 3 Department of Haemostasis and Haemostatic Disorders, Medical University of Lodz, 92-215 Lodz, Poland; cezary.watala@umed.lodz.pl; 4 Laboratory Diagnostics Unit, National Medical Institute of the Ministry of the Interior and Administration in Warsaw, 02-507 Warsaw, Poland; tomasz.korcz@cskmswia.gov.pl (T.K.); malgorzata.cicha@cskmswia.gov.pl (M.C.-B.); 5 Department of Human Epigenetics, Mossakowski Medical Research Centre Polish Academy of Sciences, 02-106 Warsaw, Poland

**Keywords:** insulin resistance, atherosclerosis, carotid intima media thickness, ankle–brachial index

## Abstract

**Background:** Insulin resistance (IR) is a condition that precedes the onset of type 2 diabetes mellitus (T2DM), which is regarded as an established risk factor for atherosclerosis (AS). Considering that the same metabolic changes as those caused by IR are evidenced to promote the development of AS, we investigated whether IR estimated by the homeostasis model assessment of IR (HOMA-IR) could predict the occurrence of preclinical AS. **Methods:** The study participants were divided into two groups based on the presence of IR diagnosed during the baseline hospitalization and defined as a HOMA-IR value equal to or higher than 2.5. After a follow-up period of at least four years, a total of 79 (*n* = 79) were prospectively assessed in terms of the presence of preclinical AS, determined by either an abnormally low ankle–brachial index (ABI) (ABI < 0.9) or an increased carotid intima media thickness (CIMT) (CIMT > 1 mm). **Results:** Using the multivariate logistic regression analysis, it was demonstrated that the HOMA-IR was associated with an abnormally low ABI (odds ratio: 1.609, 95% confidence interval (CI): [1.041–2.487], *p* = 0.032). The Cox regression model revealed that the HOMA-IR was a predictor of both an abnormal ABI (hazard ratio: 1.435, CI: [1.076–1.913], *p* = 0.014) and increased CIMT (hazard ratio: 1.419, CI: [1.033–1.948], *p* = 0.031), independently of age, sex, dyslipidemia, smoking, triglycerides (TG), low-density lipoproteins (LDL), high-density lipoproteins (HDL), and total cholesterol levels. **Conclusions:** IR, as estimated by the HOMA-IR, may be considered as a predictor of preclinical AS, independently of cardiovascular risk factors.

## 1. Introduction

Insulin resistance (IR) can be defined as an insufficient tissue response to insulin signaling [1]. The hyperinsulinemic–euglycemic clamp is considered the gold standard method of insulin sensitivity measurement. However, due to its complexity, other methods of quantifying insulin sensitivity are more frequently used in large clinical studies [2]. The homeostasis model assessment of IR (HOMA-IR) may be considered one of the most popular surrogate markers of IR; it also shows a good correlation with the hyperinsulinemic–euglycemic clamp [3].

It is estimated that the insulin-resistant state precedes the onset of type 2 diabetes mellitus (T2DM) by approximately 15 years [4]. Vascular lesions characteristic of atherosclerosis (AS) are often found in newly diagnosed diabetic individuals, which implies that the process of vascular damage begins before the onset of T2DM, during the insulin-resistant state [5,6].

Several mechanisms explaining the influence of IR on the insulin target tissues have recently been described. In the endothelium, IR affects nitric oxide (NO) production, which translates into impaired vasodilatation [7]. Hyperinsulinemia, which occurs in order to compensate for decreased insulin sensitivity, activates the mitogen-activated protein kinase pathway, which leads to increased mitogenic responsiveness to insulin [8]. Impaired vasodilatation, together with enhanced smooth muscle proliferation, contributes to atherogenic lesion formation [7].

In the adipose tissue, IR causes the inhibition of lipoprotein lipase function, which results in an increased release of free fatty acids, promoting the assembly of very low-density lipoproteins (VLDLs). The triglycerides (TGs) contained in the VLDL are transferred to either high-density lipoproteins (HDLs) or low-density lipoproteins (LDLs), forming TG-enriched HDLs or TG-enriched LDLs. The former are rapidly removed from the circulation by the kidneys, while the latter form small dense LDL-cholesterol (sdLDL) particles of enhanced atherogenic activity [7]. In hypertriglyceridemia, decreased levels of HDL cholesterol and the appearance of sdLDL are the key features of diabetic dyslipidemia and are associated with the accelerated development of AS [9].

Additionally, clinical evidence increasingly supports the link between IR and increased sympathetic nervous system activity, with significant implications for AS. IR is strongly associated with increased sympathetic nervous system activity, which contributes to both the development of atherosclerosis and the exacerbation of IR itself. Sympathetic nervous system overdrive leads to sustained hypertension through peripheral vasoconstriction, a key factor in the development and progression of atherosclerosis [10,11].

IR leads to increased plasminogen activator inhibitor 1 and fibrinogen production, enhanced thromboxane excretion, and decreased levels of tissue plasminogen activator, thereby promoting platelet aggregation and thrombosis [12]. Hyperglycemia occurs when beta-cells are no longer able to produce the sufficient quantity of insulin to compensate for impaired insulin sensitivity; in addition, free fatty acids are excessively released from the adipose tissue under the insulin-resistant state and induce oxidative stress and a proinflammatory response [4,8]. The prothrombotic state, oxidative stress, and inflammation are conditions that favor the progression of AS [8,12].

Considering the metabolic alterations caused by IR, it may be speculated that the possible causal relationship between IR and AS is likely to exist. The aim of our study was to investigate whether preclinical AS might be found in insulin-resistant patients and to assess whether IR defined as a HOMA-IR value ≥ 2.5 might predict the development of AS in the future.

## 2. Materials and Methods

### 2.1. Study Design

To conduct this study, we retrieved data from the institutional database of the National Medical Institute of the Ministry of the Interior and Administration in Warsaw. We reviewed the medical records of patients admitted to the Department of Internal Medicine, Endocrinology, and Diabetology at the National Medical Institute between 2014 and 2017. Our search specifically focused on records containing the terms: “oral glucose tolerance test”, “glucose tolerance test”, “OGTT”, and “GTT”. From these records, we identified 178 patients who met the following eligibility criteria: (1) they had undergone a prolonged (5 h) oral glucose tolerance test, (2) had complete medical records, (3) had no diagnosis of type 2 diabetes mellitus (T2DM), cancer, tumor-induced hypoglycemia, or hyperglycemia, and (4) were not taking lipid-lowering or antidiabetic medications.

At least four years after their initial hospital stay, all 178 patients were invited to participate in a prospective follow-up examination, conducted between August and September 2021. We attempted to contact all 178 patients by telephone. If a patient did not respond after three attempts, they were excluded from the study. Patients who did not attend the follow-up visit or did not provide informed consent were also excluded from further analysis. The final sample included 79 participants (*n* = 79).

The following examinations were performed both during the hospital stay (baseline) and in 2021 (follow-up visit): (1) estimation of the values of atherogenic indices (AIs), (2) calculation of the body mass index (BMI), (3) collection of blood samples and measurement of the concentration of insulin, glucose, HDL, LDL, total cholesterol, and TG, and (4) calculation of the HOMA-IR. Additionally, in order to estimate the severity of AS, all the patients that participated in the follow-up examination underwent an assessment of the ankle–brachial index (ABI) and carotid intima media thickness (CIMT). Other procedures that were performed only during the follow-up visit included measurement of waist circumference and measurement of the concentration of glycated hemoglobin (HbA1c).

The study cohort was divided into two groups based on the HOMA-IR that was calculated using the results obtained during the hospital stay between 2014 and 2017. Group 1 included patients with a HOMA-IR value lower than 2.5 (*n* = 59), while the subjects from group 2, who were considered insulin-resistant, were characterized by a HOMA-IR value equal to or higher than 2.5 (*n* = 20).

Insulin levels were measured using a noncompetitive electrochemiluminescence-based immunoassay, while blood glucose levels were estimated using the hexokinase method. HbA1c values were determined via capillary electrophoresis. The Friedewald formula was used to calculate the LDL-cholesterol levels. The HDL-cholesterol, total cholesterol, and TG levels were assessed with fluorometric–enzymatic assays.

### 2.2. Definitions

The HOMA-IR was defined as the product of fasting glucose [mg/dL] and fasting insulin concentration [uIU/mL] divided by 405 [13]. In order to recognize IR, we adopted the HOMA-IR cut-off value equal to 2.5.

The severity of subclinical AS was estimated using AIs, CIMT, and the ABI. We calculated the values of the AIs using the following equations: (1) atherogenic index of plasma (AIP): log10(TG/HDL-cholesterol); (2) Castelli’s risk index I (CRI-I): total cholesterol/HDL-cholesterol; (3) Castelli’s risk index II (CRI-II): LDL-cholesterol/HDL-cholesterol; and (4) atherogenic coefficient (AC): non-HDL-cholesterol/HDL-cholesterol [14].

The ABI is one of the tools that enables the assessment of vascular impairment present in the peripheral arteries [15]. According to the scientific statement from the American Heart Association, the ABI may be regarded as a good marker of peripheral artery disease (PAD), with acceptable sensitivity and specificity [16]. In our study, the ABI was defined as the highest of the systolic blood pressure (SBP) values measured on the dorsalis pedis and posterior tibial artery divided by the highest of the left and right brachial SBP values [17]. SBP was assessed with the employment of a pocket blood flow detector (Sonomed Doppler MD4, Sonomed Ltd., Warsaw, Poland). An ABI value lower than 0.9 was considered a marker of PAD and preclinical AS [14], while an ABI higher than 1.4 was classified as an indicator of medial arterial calcification [17].

CIMT was assessed following the recommendations of the American Society of Echocardiography [18]. All the study participants were examined with the same Canon Aplio a450 ultrasound scanner, TMS Ltd., Warsaw, Poland and by the same operator. In order to estimate CIMT, B-mode ultrasound images of the distal 10 mm of the distal wall of the common carotid artery were obtained bilaterally. The mean value of CIMT was calculated on the basis of a three-angle CIMT measurement on each side. The higher of the mean right and mean left CIMT values was used in the further analysis. Results higher than 1 mm were considered as abnormal [19].

The following diagnostic criteria for dyslipidemia were applied: TG ≥ 150 mg/dL, or TC ≥ 190 mg/dL, or LDL-cholesterol ≥ 115 mg/dL, or HDL-cholesterol < 40 mg/dL (for men) and <45 mg/dL (for women) [20].

### 2.3. Statistical Analysis

The descriptive data are shown as means + standard deviation or as medians with interquartile ranges. Categorical variables were compared using the chi-square test. Comparisons between normally distributed continuous variables were performed using the Student’s *t*-test. Non-normally distributed continuous data were analyzed using the Mann–Whitney U test. We used multivariate logistic regression to assess the relationship between the HOMA-IR and the markers of preclinical AS, upon the adjustment to a set of the confounding variables of choice. In the time-dependent analysis, the potential of the HOMA-IR, BMI, age, and other selected confounding variables to predict the occurrence of preclinical AS during an observation period of at least four years was estimated using the Cox proportional hazard regression analysis. Both the multivariate and Cox regression analyses were adjusted for the following confounders: age, sex, BMI, smoking (at present and/or in the past), the HOMA-IR, dyslipidemia, and serum concentrations of TG, HDL, LDL, and total cholesterol. The goodness of fit of the logistic regression models showing a significant discrimination between controls and patients was estimated using the Hosmer–Lemeshow test. In some bivariate and multivariate analyses, we used the approach of resampling with replacement (the bootstrap-boosted versions of the tests, with 10,000 iterations) to make sure that the revealed differences were not detected by pure chance. The borderline of the significance level was accepted at *p* < 0.05. The collected data were analyzed using the Statistica 13.3 software (StatSoft Polska Sp. z. o. o. 2022) and R Package Software v. 4.4.

## 3. Results

### 3.1. Study Group Characteristics

The clinical and biochemical characteristics of the study participants are given in Table 1. Additionally, a comparison of baseline characteristics between patients included in the follow-up study and those excluded is available in the Supplementary Material (Table S1).

At the baseline, there were no statistical differences between group 1 (the patients without IR) and group 2 (the insulin-resistant group) in terms of age (37.8 ± 13.4 y vs. 34.4 ± 11.8 y; *p* = 0.272), gender (9 (15.25%) vs. 6 (30%) male patients; *p* = 0.167), prevalence of arterial hypertension (10 (16.95%) vs. 4 (20%) patients; *p* = 0.876), and dyslipidemia (19 (32.2%) vs. 6 (30%) patients; *p* = 0.784). At the baseline, no patient was on lipid-lowering medication or on antihyperglycemic therapy. During the follow-up visit, two patients from group 1 (3.39%) and one patient from group 2 (5%) reported taking statins, while nine subjects from group 1 (15.25%) and nine individuals from group 2 (45%) were taking metformin. No major adverse cardiovascular events defined as an occurrence of myocardial infarction, stroke, or cardiovascular death were reported during the follow-up period. During a four-year observation period, eleven (18.64%) subjects from group 1 developed insulin resistance, while four (20%) patients from group 2 became non-insulin-resistant. Two patients from group 1 and no patients from group 2 developed T2DM.

The laboratory results showed that the patients with insulin resistance had significantly higher baseline markers of glucose metabolism, including fasting glucose levels and mean fasting insulin concentrations, compared to those without insulin resistance. Additionally, the mean fasting insulin concentration remained significantly higher in the insulin-resistant group during the follow-up visit. (Table 1).

There were significant differences between the groups in terms of adiposity markers. The insulin-resistant patients had significantly higher baseline BMIs than the individuals without IR (Table 1). The mean waist circumference measured during the follow-up appointment was also significantly higher in the patients from the insulin-resistant group (94.3 ± 18.9 cm vs. 104 ± 15.6 cm; *p* = 0.021).

No significant difference between the groups was observed in terms of total and LDL cholesterol concentration, regardless of the time when the lipid profile was assessed. Conversely, the mean HDL cholesterol concentration was significantly higher in the first group, both during the first hospitalization and at the follow-up visit (Table 1).

Irrespective of the time when the results were obtained, the values of CRI-I, CRI-II, and AC were significantly higher in the insulin-resistant group. Although the results obtained during hospitalization showed that the value of the AIP was significantly higher in the insulin-resistant group, no significant difference was found between the follow-up results of the group without IR compared to the results of the group with IR (Table 1).

### 3.2. ABI and CIMT

There was no statistically significant difference between the groups in terms of the mean ABI (Table 2). Similarly, a comparison between the analyzed groups regarding the number of individuals with abnormal ABIs revealed no differences (Table 2). The mean CIMT measured in the non-insulin-resistant subjects was comparable to that measured in the insulin-resistant patients. The prevalence of abnormal CIMT was similar in the two groups (Table 2).

### 3.3. Association Between HOMA-IR, BMI, and Markers of Preclinical AS

Bootstrapped multivariate logistic regression showed that the HOMA-IR was associated with preclinical AS defined as an ABI lower than 0.9, independently of age, sex, dyslipidemia, smoking, triglycerides, LDL-, HDL- and total cholesterol levels. Contrary to our expectations, the BMI was found to be associated with a decreased risk of an abnormally low ABI (Table 3). In the crude model, we observed that the BMI was significantly associated with the increased CIMT. This relationship remained significant even after adjusting for other cardiovascular risk factors (Table 4).

The Cox regression analysis revealed that the HOMA-IR was a predictor of preclinical AS determined by either increased CIMT or abnormal ABI values, independently of the analyzed confounding factors (Table 5 and Table 6). We found that the BMI was significantly associated with increased CIMT. Interestingly, the patients with a higher BMI were less likely to have an abnormally low ABI (Table 5 and Table 6).

## 4. Discussion

It is well established that there is an association between atherosclerosis (AS) and metabolic syndrome, yet the specific role of individual factors involved in the shared pathophysiology of these conditions remains challenging to determine [21]. Insulin resistance (IR) is a key component of metabolic syndrome, and several mechanisms may explain its link to AS [22]. One major mechanism involves the insulin resistance-induced reduction in nitric oxide (NO) bioavailability, which is essential for maintaining endothelial function [23]. Reduced NO levels contribute to atherosclerotic plaque formation by promoting platelet aggregation, enhancing monocyte adhesion, and increasing the infiltration of vascular smooth muscle cells into the endothelium, all of which play a central role in AS development [24]. Clinical evidence suggests that targeting asymmetric dimethylarginine, an endogenous inhibitor of NO, may offer a promising therapeutic strategy for cardiovascular disease, as NO deficiency is implicated in its pathogenesis [25].

Additionally, the sympathetic nervous system has been shown to contribute to the pathophysiology of both AS and IR. Studies suggest that hyperinsulinemia, a key marker of IR, activates the sympathetic nervous system, while recent research proposes that sympathetic nervous system overstimulation may actually drive hyperinsulinemia through reduced blood flow to muscles [10]. This increased sympathetic activity contributes to hypertension, which is an established risk factor for AS [11]. Emerging clinical reports on multi-organ denervation demonstrate its ability to reduce sympathetic nerve activity, decrease atheroma size, and improve insulin sensitivity. These findings underscore the potential benefits of targeting both IR and AS in the same therapeutic intervention [26,27]. A deeper understanding of the relationship between AS and IR could offer significant clinical benefits, including the development of more effective treatments and improved outcomes for patients with these interconnected conditions. Providing new evidence on the clinical significance of the IR-AS relationship could help determine whether there is a rationale for intensifying clinical interventions to detect and treat IR.

Our results revealed that compared to the non-insulin-resistant subjects, the patients with IR had significantly higher markers of adiposity, namely the baseline BMI and follow-up BMI (Table 1). The authors of the ESC guidelines on cardiovascular disease (CVD) prevention enlisted adiposity as one of the major modifiable AS CVD risk factors. A meta-analysis of 58 prospective studies on the association of adiposity measures with CVD demonstrated that both BMI and waist circumference were strongly and similarly associated with AS CVD [21]. In our study, the patients with IR were characterized by significantly higher baseline BMIs and body mass values and significantly higher waist circumference than the individuals without IR. Similar findings were reported by Caporaso et al., who investigated a relationship between IR and health-related outcomes in a large cohort of generally heathy subjects. They found that waist circumference and BMI values increased across the increasing quartiles of the HOMA-IR. This trend persisted even after adjusting for confounding variables, such as age, social status, ethnicity, gender, and smoking and drinking habits, irrespective of the way the HOMA-IR was estimated (as the median, quartiles, or a continuous variable) [22].

In our study, patients with IR had significantly lower baseline and follow-up HDL cholesterol levels and significantly higher baseline TG levels than the individuals without IR, with the mean TG levels exceeding the threshold of 150 mg/dL (Table 1). TG level assessment is recommended to identify individuals in whom LDL cholesterol levels may underestimate the AS CVD risk and is to be performed routinely during the standard lipid profile assessment [20]. While TG levels higher than 150 mg/dL were shown to be associated with AS development, high HDL cholesterol levels were inversely associated with CVD risk [20]. Our results suggest that the patients with IR were characterized by a combination of lipid abnormalities, which are also regarded AS risk factors. Such findings are not surprising provided that multiple studies demonstrated that diabetic dyslipidemia, defined as increased TG and sdLDL cholesterol levels and reduced HDL cholesterol levels, is commonly confirmed in insulin-resistant patients [23,28]. IR is considered a major trigger for diabetic dyslipidemia as it facilitates an increased secretion and decreased clearance of VLDL, which results in hypertriglyceridemia [7]. Furthermore, IR is associated with increased hepatic lipase activity and an enhanced production of TG-enriched HDL particles, which leads to increased HDL catabolism and, thus, decreased HDL levels, which we observed in the insulin-resistant group [7,23].

Numerous authors reported that AIs were strongly associated with AS and might represent a useful tool for AS risk stratification [14,29,30]. Our results (Table 1) suggest that insulin-resistant patients were characterized by significantly higher AI values than the non-insulin-resistant subjects (Table 1). Similar findings were reported by Du et al., who found that insulin-resistant patients were more likely to have significantly higher values of CRI-I, CRI-II, AC, and the TG/HDL ratio than the subjects without IR. Furthermore, they demonstrated a strong association between each of the lipid ratios and IR [31]. The adjusted analysis conducted by Caporaso et al. revealed that the TG to HDL cholesterol ratio tended to increase along with increasing HOMA-IR quartiles [22]. Instead of the crude TG/HDL ratio, we measured AIP, which is a logarithmically transformed TG/HDL ratio. Nevertheless, our results are consistent with both of the aforementioned studies and indicate that patients with IR have significantly higher AIs, which may be regarded as a marker of preclinical AS [29,30].

Our results indicate that the HOMA-IR was associated with markers of preclinical AS independently of cardiovascular risk factors and may be regarded as an independent predictor of abnormal CIMT (Table 4). These findings are consistent with the results of the IRAS study (the Insulin Resistance Atherosclerosis Study), which was the first large, epidemiologic study on the association between insulin sensitivity and prevalent CVD. The IRAS study revealed an inverse association between insulin sensitivity and AS, as estimated by CIMT in the group of Caucasian individuals, which persisted even after adjustment for the established CVD risk factors [32]. In contrast, the results of the MESA study revealed that the relationship between IR and CIMT could not be considered independent as it lost its significance after adjusting for the metabolic syndrome components. Based on those findings, the authors of the MESA study did not recommend the use of the HOMA-IR as an additional AS risk assessment tool as it did not seem to improve CV risk stratification [33]. Like the results of the IRAS study, a meta-analysis by Gast et al., and the Bruneck Study, our results are in opposition to those conclusions and support the hypothesis that the inclusion of the HOMA-IR in the AS risk prediction model may be beneficial [32,34,35].

While numerous authors reported on the association between the ABI and DM, CVD, and metabolic syndrome, little is known about the relationship between the ABI and IR [36]. Britton et al. were the first authors to conduct a prospective analysis of the association between IR and PAD, defined as either an ABI < 0.9 or the development of clinical PAD during the time of observation [36]. They confirmed an independent association between the HOMA-IR and the ABI, and the correlation remained significant even after diabetic patients were excluded from the analysis [36]. Despite the fact that our study was conducted only in Caucasian individuals with a mean age of 36.9 years old, we observed associations (Table 3 and Table 5) that were similar to those reported by Britton et al. in 2012 (85% Caucasian patients, mean age: 72.3 years old).

Recent studies regarding the relationship between BMI and CIMT have brought contradictory results. Our findings (Table 6), which are similar to those of Bretton et al. (2011), revealed that BMI was significantly associated with CIMT [37]. As demonstrated by Landecho et al. (2018) and Ge et al. (2014), other markers of obesity, such as high body fat percentage and increased waist circumference, tended to present a higher magnitude of association with CIMT than BMI [38,39].

Although obesity is considered a major risk factor for PAD, we found that BMI was inversely associated with an abnormally low ABI (Table 3 and Table 5) [40]. The positive association between BMI and arterial stiffness and, thus, a falsely high ABI, could explain our results [41]. However, considering the recently published studies reporting divergent results regarding the association between obesity and arterial stiffness, this relationship seems to be more complex [42]. Therefore, further research in this field is needed.

Our study has several limitations. In the present study, the method of IR estimation was different from the gold standard method, i.e., the glucose clamp test [3]. As we were aware that it is not used as frequently as other IR assessment tools due to its invasiveness and complexity, we decided to employ the HOMA-IR [3]. Although the HOMA-IR is a surrogate marker of IR, it shows a good correlation with the glucose clamp test [3]. Regrettably, no definite guidelines on how to diagnose IR using indirect IR indices have been introduced yet, nor have any cut-off values been established [3]. The threshold of 2.5 that was applied in this study is also that which is most frequently used in the literature [3]. Since our study was of a cross-sectional nature, no cause–effect relationship could be determined.

## 5. Conclusions

In conclusion, we found that patients with IR, defined as a HOMA-IR value ≥ 2.5, were characterized by significantly higher AI values and TG levels and significantly lower HDL cholesterol levels than the individuals without IR. Using both the multivariate logistic regression model and Cox regression analysis, we demonstrated that the HOMA-IR can be considered as an independent predictor of an abnormal ABI. Furthermore, we found that the HOMA-IR was significantly associated with increased CIMT, independently of age, sex, arterial, dyslipidemia, smoking, LDL-, HDL- and total cholesterol and triglyceride levels. Therefore, our results suggest that the HOMA-IR can be regarded as an independent predictor of preclinical AS. Further research is necessary to determine whether there are implications for a more aggressive approach towards AS prevention in patients with IR. Large-cohort, longitudinal studies are needed to establish whether IR may be considered an AS risk factor.

## Figures and Tables

**Table 1:** Patient characteristics.

	Baseline			Follow-Up		
Variables	Group 1 (*n* = 59)	Group 2 (*n* = 20)	*p*	Group 1 (*n* = 59)	Group 2 (*n* = 20)	*p*
BMI [kg/m^2^]	28.5 ± 7.5	32.5 ± 5.6	0.01 *	27.2 ± 6.9	29.9 ± 6.1	0.08
Fasting glucose [mg/dL]	82.7 ± 8.8	88.1 ± 6.1	0.01 *	93.1 ± 15.5	94.2 ± 10.7	0.32
IFG (*n*) [%]	2 (3.39%)	0	0.82	13 (22.03%)	4 (20%)	0.86
Fasting insulin [mIU/L]	6.8 ± 2.95	19.6 ± 9.33	0.000001 *	10.6 ± 7.5	21.9 ± 11.6	0.00002 *
AIP	0.18 ± 0.3	0.5 ± 0.3	0.001 *	0.21 ± 0.3	0.32 ± 0.34	0.3
CRI-I	3.11 ± 1.05	4.25 ± 1.5	0.0009 *	3.2 ± 1.02	3.99 ± 1.75	0.04 *
CRI-II	1.7 ± 0.8	2.52 ± 1.03	0.002 *	1.75 ± 0.76	2.23 ± 0.9	0.03 *
AC	2.11 ± 1.05	3.25 ± 1.5	0.001 *	2.17 ± 1.02	2.99 ± 1.74	0.04 *
Total cholesterol [mg/dL]	180.7 ± 29.2	189.1 ± 37.3	0.44	190.7 ± 31.2	201.2 ± 50.9	0.8
LDL cholesterol [mg/dL]	96.6 ± 26.5	111.2 ± 29.5	0.06	103.4 ± 28.1	114.6 ± 37.8	0.33
HDL cholesterol [mg/dL]	63.1 ± 18.9	47.8 ± 13.6	0.003 *	64.6 ± 18.3	55.7 ± 21.1	0.01 *
Triglycerides [mg/dL]	105.9 ± 63.5	155.8 ± 88.9	0.004 *	117.6 ± 62.7	143.2 ± 146.5	0.72

IFG—impaired fasting glucose; AIP—atherogenic index of plasma; CRI-I—Castelli’s risk index I; CRI-II—Castelli’s risk index II; AC—atherogenic coefficient; LDL—low-density lipoprotein; HDL—high-density lipoprotein, *—statistically significant.

**Table 2:** Results from the follow-up examination: assessment of the markers of preclinical atherosclerosis.

Variables	Group 1—Follow-Up (*n* = 59)	Group 2—Follow-Up (*n* = 20)	*p*
ABI mean	1.08 ± 0.12	1.18 ± 0.24	0.161
ABI < 0.9 [*n* (%)]	13 (22.03%)	5 (25%)	0.84
ABI > 1.4 [*n* (%)]	0	2 (10%)	0.51
Mean CIMT [mm] follow-up	0.75 ± 0.25	0.78 ± 0.21	0.35
CIMT > 0.8 mm [*n* (%)] follow-up	17 (28.81%)	7 (35%)	0.68
CIMT > 1 mm [*n* (%)] follow-up	10 (16.95%)	6 (30%)	0.39

ABI—ankle–brachial index; CIMT—carotid intima media thickness.

**Table 3:** Relationships between insulin resistance (IR) and the presence of preclinical atherosclerosis determined by abnormally low ankle–brachial index (ABI) in groups of patients showing abnormally low ABI (marked ABI < 0.9) and controls (ABI ≥ 0.9).

Variable/Risk Factor	Control (ABI ≥ 0.9) (*n* = 61)		Low-ABI Patients (ABI < 0.9) (*n* = 18)		Crude OR (95% CI)	*p*	Adjusted OR (95% CI) *	*p*
	Median (IQR) Number/Frequency		Median (IQR) Number/Frequency					
Explanatory variables:								
Insulin resistance (HOMA-IR)	1.66 (1.01–2.50)		1.53 (0.96–2.56)		1.050 (0.789–1.398)	0.739	**1.609 (1.041–2.487)** **^&^** **1.697 (1.068–2.696)**	**0.032** **0.025**
BMI [kg/m^2^]	31.1 (24.4–36.3)		26.5 (22.5–29.4)		**0.896 (0.812–0.988)** **^&^** **0.894 (0.821–0.972)**	**0.027** **0.009**	**0.594 (0.423–0.833)** **^&^** **0.578 (0.378–0.884)**	**0.003** **0.011**
Confounding variables:								
Age [yr]	36.0 (26.3–44.8)		31.5 (27.5–41.8)		0.987 (0.945–1.029)	0.534		
Sex [male]	11	18.6	4	22.2	1.247 (0.343–4.529)	0.538		
Smoking [0/1]	10	16.9	4	22.2	1.400 (0.380–5.152)	0.613	1.562 (0.393–6.199)	0.526
Smoking at present [0/1]	13	22.4	3	16.7	0.692 (0.173–2.765)	0.603	0.582 (0.140–2.417)	0.457
Dyslipidemia [0/1]	19	32.2	6	33.3	1.053 (0.343–3.232)	0.929	1.163 (0.352–3.843)	0.805
Total cholesterol [mg/dL]	186 (159–207)		163 (145–210)		0.988 (0.970–1.006)	0.200	0.987 (0.967–1.008)	0.219
LDL cholesterol [mg/dL]	102 (86–129)		91 (61–112)		0.980 (0.959–1.001) ^&^ 0.979 (0.955–1.005)	0.058 0.111	0.978 (0.956–1.000) ^&^ 0.976 (0.946–1.008)	0.054 0.143
HDL cholesterol [mg/dL]	58 (45–72)		56 (47–71)		0.999 (0.970–1.028)	0.939	1.001 (0.971–1.032)	0.938
Triglycerides [mg/dL]	105 (72–137)		84 (63–166)		1.005 (0.998–1.011)	0.196	0.989 (0.946–1.033)	0.610

Continuous variables given as medians and interquartile ranges; categorical ones—as numbers and frequencies. * OR, presented as OR (±95% CI), calculated with the aid of multiple logistic regression analysis; crude OR values are adjusted for all (presented in the table above) confounding variables in the case of HOMA-IR and BMI, or adjusted for age and sex in the case of all the remaining confounders. *p* < 0.05 and the corresponding ORs are in bold. ^&^ The bootstrap-boosted OR values, estimated along with the classical resampling procedure with 10,000 iterations, are given for statistically significant outcomes.

**Table 4:** Relationships between insulin resistance (IR) and the presence of preclinical atherosclerosis determined by increased carotid intima media thickness (CIMT) in groups of patients showing increased CIMT (marked (CIMT > 1 mm) and controls (CIMT ≤ 1 mm).

Variable/Risk Factor	Control (CIMT ≤ 1 mm) (*n* = 63)		Increased-CIMT Patients (CIMT > 1 mm) (*n* = 16)		Crude OR (95% CI)	*p*	Adjusted OR (95% CI) *	*p*
	Median (IQR) Number/Frequency		Median (IQR) Number/Frequency					
Explanatory variables:								
Insulin resistance (HOMA-IR)	1.46 (0.96–2.42)		2.07 (1.55–2.42)		**1.366 (1.005–1.858)** **^&^** 1.390 (0.986–0.960)	**0.047** 0.060	2.492 (0.787–7.893) ^&^ 2.348 (0.781–7.057)	0.121 0.129
BMI [kg/m^2^]	27.1 (22.7–32.1)		33.5 (28.9–37.9)		**1.129 (1.036–1.230)** **^&^ 1.137 (1.035–1.249)**	**0.006** **0.0007**	**1.587 (1.082–2.328)** **^&^ 1.616 (1.067–2.449)**	**0.018** **0.024**
Confounding variables:								
Age [yr]	31.0 (25.0–41.0)		51.0 (41.5–59.0)		**1.107 (1.048–1.169)** **^&^ 1.113 (1.050–1.179)**	**0.0003** **0.0003**		
Sex [male]	9	14.8	6	37.5	**3.467 (1.008–11.919)** ^&^ 3.437 (0.730–16.180	**0.049** 0.118		
Smoking [0/1]	8	13.1	6	37.5	**3.975 (1.132–13.955)** **^&^ 3.637 (1.049–12.611)**	**0.031** **0.042**	2.233 (0.526–9.471)	0.276
Smoking at present [0/1]	13	21.7	3	18.8	0.834 (0.206–3.375)	0.800	1.004 (0.202–5.001)	0.996
Dyslipidemia [0/1]	16	26.2	9	56.3	**3.616 (1.156–11.314)** **^&^ 3.437 (1.169–10.108**	**0.027** **0.024**	1.788 (0.452–7.077)	0.408
Total cholesterol [mg/dL]	173 (152–204)		206 (193–214)		**1.035 (1.012–1.057)** **^&^ 1.037 (1.014–1.060)**	**0.002** **0.001**	1.022 (0.997–1.049)	0.088
LDL cholesterol [mg/dL]	91 (76–113)		125 (106–136)		**1.047 (1.018–1.076)** **^&^** 1.049 (1.022–1.077)	**0.001** **0.0004**	**1.040 (1.006–1.075)** **^&^ 1.046 (1.003–1.091)**	**0.022** **0.035**
HDL cholesterol [mg/dL]	59 (46–72)		50 (44–63)		0.993 (0.963–1.024)	0.654	0.964 (0.919–1.010)	0.125
Triglycerides [mg/dL]	90 (66–137)		123 (109–144)		1.003 (0.996–1.010)	0.393	1.003 (0.994–1.012)	0.539

Continuous variables given as medians and interquartile ranges; categorical ones—as numbers and frequencies. * OR, presented as OR (±95% CI), calculated with the aid of multiple logistic regression analysis; crude OR values are adjusted for all (presented in the table above) confounding variables in the case of HOMA-IR and BMI, or adjusted for age and sex in the case of all the remaining confounders. *p* < 0.05 and the corresponding ORs are in bold. ^&^ The bootstrap-boosted OR values, estimated along with the classical resampling procedure with 10,000 iterations, are given for statistically significant outcomes.

**Table 5:** Time-dependent relationship between insulin resistance (IR) and the presence of preclinical atherosclerosis determined by abnormally low ankle–brachial index (ABI) in groups of patients showing abnormally low ABI (marked ABI < 0.9) and controls (ABI ≥ 0.9).

Variable/Risk Factor	Crude HR (±95% CI)	*p*	Adjusted HR (±95% CI) *	*p*
Explanatory variables:				
Insulin resistance (HOMA-IR)	1.101 (0.885–1.370)	0.387	**1.435 (1.076–1.913)** **^&^ 1.417 (1.081–1.857)**	**0.014** **0.011**
BMI [kg/m^2^]	0.929 (0.856–1.008)	0.075	**0.701 (0.543–0.904)** **^&^ 0.706 (0.549–0.908)**	**0.006** **0.007**
Confounding variables:				
Age [yr]	0.984 (0.948–1.020)	0.371		
Sex [male]	0.788 (0.259–2.393)	0.674		
Smoking [0/1]	0.955 (0.308–2.959)	0.936	0.760 (0.224–2.576)	0.660
Smoking at present [0/1]	1.489 (0.429–5.174)	0.531	1.893 (0.497–7.206)	0.349
Dyslipidemia [0/1]	1.330 (0.489–3.620)	0.576	1.143 (0.382–3.416)	0.811
Total cholesterol [mg/dL]	0.989 (0.973–1.006)	0.207	0.990 (0.972–1.008)	0.287
LDL cholesterol [mg/dL]	0.982 (0.964–0.999) ^&^ 0.963 (0.926–1.002)	0.049 0.061	0.981 (0.962–1.001) ^&^ 0.983 (0.958–1.008)	0.054 0.178
HDL cholesterol [mg/dL]	1.001 (0.976–1.028)	0.911	1.006 (0.978–1.035)	0.680
Triglycerides [mg/dL]	1.003 (0.998–1.009)	0.214	1.004 (0.998–1.009)	0.205

* HR, presented as HR (±95% CI), calculated with the aid of Cox proportional hazard regression analysis; crude HR values are adjusted for all (presented in the table above) confounding variables in the case of HOMA-IR and BMI, or adjusted for age and sex in the case of all the remaining confounders. *p* < 0.05 and the corresponding HRs are in bold. ^&^ The bootstrap-boosted HR values, estimated along with the classical resampling procedure with 10,000 iterations, are given for statistically significant outcomes.

**Table 6:** Time-dependent relationship between insulin resistance (IR) and the presence of preclinical atherosclerosis determined by increased carotid intima media thickness (CIMT) in groups of patients showing increased CIMT (marked (CIMT > 1 mm) and controls (CIMT ≤ 1 mm).

Variable/Risk Factor	Crude HR (±95% CI)	*p*	Adjusted HR (±95% CI) *	*p*
Explanatory variables:				
Insulin resistance (HOMA-IR)	**1.245 (1.052–1.473)** **^&^ 1.242 (1.042–1.479)**	**0.011** **0.016**	**1.419 (1.033–1.948)** **^&^ 1.421 (1.020–1.981)**	**0.031** **0.038**
BMI [kg/m^2^]	**1.091 (1.023–1.164)** **^&^ 1.091 (1.006–1.183)**	**0.008** **0.035**	**1.280 (1.078–1.519)** **^&^ 1.277 (1.071–1.522)**	**0.005** **0.006**
Confounding variables:				
Age [yr]	**1.049 (1.015–1.085)** **^&^** **1.044 (1.005–1.084)**	**0.005** **0.027**		
Sex [male]	0.385 (0.140–1.060)	0.065		
Smoking [0/1]	0.509 (0.176–1.472)	0.213	0.855 (0.266–2.752)	0.793
Smoking at present [0/1]	1.312 (0.371–4.632)	0.673	1.287 (0.341–4.858)	0.710
Dyslipidemia [0/1]	0.601 (0.211–1.713)	0.341	1.068 (0.347–3.287)	0.909
Total cholesterol [mg/dL]	**1.025 (1.007–1.043)** **^&^ 1.023 (1.004–1.043)**	**0.006** **0.018**	1.012 (0.993–1.031)	0.227
LDL cholesterol [mg/dL]	**1.032 (1.008–1.056)** **^&^ 1.036 (1.006–1.067)**	**0.008** **0.019**	1.021 (0.997–1.045)	0.083
HDL cholesterol [mg/dL]	0.999 (0.972–1.026)	0.942	0.983 (0.952–1.015)	0.299
Triglycerides [mg/dL]	1.002 (0.996–1.009)	0.438	1.002 (0.995–1.009)	0.540

* HR, presented as HR (±95% CI), calculated with the aid of Cox proportional hazard regression analysis; crude HR values are adjusted for all (presented in the table above) confounding variables in the case of HOMA-IR and BMI, or adjusted for age and sex in the case of all the remaining confounders. *p* < 0.05 and the corresponding HRs are in bold. ^&^ The bootstrap-boosted HR values, estimated along with the classical resampling procedure with 10,000 iterations, are given for statistically significant outcomes.

## Data Availability

The datasets used and analyzed during the current study are available from the corresponding author on reasonable request.

## Supplementary Materials

The following supporting information can be downloaded at: https://www.mdpi.com/article/10.3390/jcm14030969/s1, Table S1: A comparison between the population included in the follow-up study (group 2) and the patients excluded from the follow-up (group 1).

## Abbreviations

ABI, ankle–brachial index; AC, atherogenic coefficient; AIs, atherogenic indices; AIP, atherogenic index of plasma; AS, atherosclerosis; BMI, body mass index; CI, confidence interval; CIMT, carotid intima media thickness; CRI-I, Castelli’s risk index I; CRI-II, Castelli’s risk index II; CVD, cardiovascular disease; HbA1c, glycated hemoglobin; HDL, high-density lipoprotein; HOMA-IR, homeostasis model assessment of IR; HR, hazard ratio; IFG, impaired fasting glucose; IR, insulin resistance; LDL, low-density lipoprotein; NO, nitric oxide; OR, odds ratio; PAD, peripheral artery disease; sdLDL, small dense LDL-cholesterol; SBP, systolic blood pressure; T2DM, type 2 diabetes mellitus; TG, triglycerides; VLDL, very low-density lipoproteins
