# Supplementary material for: Is Insulin Resistance an Independent Predictor of Atherosclerosis?
Source: J Clin Med. 2025 Feb 3;14(3):969. doi: 10.3390/jcm14030969 (PMC11818120; doi:10.3390/jcm14030969)
Supplement: Supplementary file 1 [file jcm-14-00969-s001.zip › jcm-3412931-supplementary.pdf]

Table S1. A comparison between the population included in the follow-up study (group 2) and the patients excluded from the follow-up (group 1).

Variables	Group 1 (*n* =99)	Group 2 (*n* = 79)	p
BMI [kg/m²]	28.30±8.43	29.36±7.22	0.16
Fasting glucose [mg/dL]	84.64±9.87	83.94±8.45	0.79
IFG (n) [%]	7 (7.07)	2 (2.53)	0.17
Fasting insulin [mIU/L]	10.58±8.11	10.15±7.75	0.74
AIP	0.19±0.32	0.26±0.33	0.19
CRI-I	3.20±1.27	3.36±1.26	0.44
CRI-II	1.73±0.95	1.87±0.95	0.35
AC	2.20±1.27	2.36±1.26	0.44
Total cholesterol [mg/dL]	179.67±39.81	181.66±32.08	0.73
LDL cholesterol [mg/dL]	97.38±35.41	99.21±28.71	0.61
HDL cholesterol [mg/dL]	61.89±19.15	59.49±18.67	0.42
Triglycerides [mg/dL]	105.49±62.83	117.80±72.35	0.18

BMI – body mass index; IFG—impaired fasting glucose; AIP—atherogenic index of plasma; CRI-I—Castelli's risk index I; CRI-II—Castelli's risk index II; AC—atherogenic coefficient; LDL—low-density lipoprotein; HDL—high-density lipoprotein.
